# Transarterielle periartikuläre Embolisation (TAPE) – Vom Schmerz zur Stille: TAPE als neue lokale Option bei Arthrose und perspektivisch bei entzündlich rheumatischen Gelenkerkrankungen

**DOI:** 10.1007/s00393-025-01708-x

**Published:** 2025-08-21

**Authors:** Alexander Pfeil, Carolin Pflug, Gunter Wolf, Ulf Teichgräber, René Aschenbach

**Affiliations:** 1 Rheumazentrum (G-BA-Kriterien) sowie Sektion Rheumatologie und Osteologie, Klinik für Innere Medizin III, Universitätsklinikum Jena, Am Klinikum 1, 07747 Jena, Deutschland; 2 Institut für Diagnostische und Interventionelle Radiologie, Universitätsklinikum Jena, Jena, Deutschland

**Keywords:** Transarterielle periartikuläre Embolisation (TAPE), Entzündliche rheumatische Gelenkerkrankungen, Arthrose, Monarthritis, Checkpointinhibitor-assoziierte Arthritis, Transarterial periarticular embolization (TAPE), Inflammatory rheumatic joint diseases, Osteoarthritis, Monarthritis, Checkpoint inhibitor associated arthritis

## Abstract

Die transarterielle periartikuläre Embolisation (TAPE) stellt ein neuartiges interventionelles Verfahren zur lokalen Schmerztherapie bei Arthrose dar. Die Reduktion der lokalen Hypervaskularisation wird durch die supraselektive Embolisation periartikulärer Arterien mit geeigneten Embolisaten erreicht. Das beschriebene Verfahren resultiert in einer Reduktion sowohl der Schmerzen als auch der Einnahme von Analgetika. Ursprünglich zur Behandlung der Gonarthrose entwickelt, zeigen erste kleine Studien und Fallberichte vielversprechende Ergebnisse auch bei anderen Gelenken wie Fingern und Schulter. In der Rheumatologie eröffnet die TAPE insbesondere bei therapieresistenter Monarthritis sowie bei Checkpointinhibitor-assoziierter Arthritis neue therapeutische Perspektiven.

## Abstract (en)

Transarterial periarticular embolization (TAPE) is a novel interventional procedure for local pain therapy in osteoarthritis. The reduction of local hypervascularization is achieved by supraselective embolization of periarticular arteries using embolic agents. The procedure described herein results in a reduction in both pain and the use of analgesics. The drug was originally developed for the treatment of knee osteoarthritis and initial small studies and case reports indicated that it was effective for other joints, including the fingers and shoulders. In the domain of rheumatology TAPE opens up new therapeutic perspectives, particularly for cases of treatment-resistant monoarthritis and arthritis associated with checkpoint inhibitors.

Zur Behandlung von Arthrosen stehen operative und konservative Therapieverfahren zur Verfügung [11]. Sicherlich gilt im Bereich der Hüft- und Kniegelenke der operative Kniegelenkersatz als Goldstandard, aber nicht jeder Patient profitiert von einem endoprothetischen Gelenkersatz, insbesondere unter Berücksichtigung von Komorbiditäten [7]. Weiterführend stellt der Arthroseschmerz trotz umfassender konservativer Optionen eine klinische Herausforderung dar – insbesondere bei Patient:innen mit Kontraindikationen (z. B. chronische Niereninsuffizienz) für systemische Analgetikatherapien oder fortbestehenden Beschwerden unter einer Standardtherapie.

In diesem Kontext ist die *transarterielle periartikuläre Embolisation* (TAPE) als neues Verfahren zur Behandlung der Arthrose (insbesondere der Gonarthrose) einzuordnen. Im Rahmen dieses Übersichtsartikels soll das Verfahren der TAPE vorgestellt werden und ein Ausblick bezüglich des Einsatzes im Rahmen von entzündlich rheumatischen Gelenkerkrankungen gegeben werden.

## TAPE – Technik und Wirkprinzip

Die TAPE ist ein minimal-invasives interventionelles Verfahren, das über eine arterielle Gefäßpunktion mittels Kontrastmittelapplikation in der digitalen Subtraktionsangiographie zunächst die Gefäßanatomie des zu behandelnden Gelenks dargestellt [10].

Typischerweise findet sich ein hypervaskularisiertes Gewebemuster in den betroffenen Gelenkabschnitten, welches „wolkenartig“ als sog. Blush darstellbar ist und Zonen der Neovaskularisierung entspricht. Unter bildgebender Kontrolle werden die betroffenen periartikulären Arterien durch einen Mikrokatheter supraselektiv sondiert. Anschließend wird ein Embolisat appliziert, um den Blush auszuschalten [10]. Zur Embolisation werden in der Regel temporäre Embolisate wie Cilastatin/Imipenem als auch resorbierbare Partikel eingesetzt, die zu einem vorübergehenden Verschluss der kleinen Arterien führen [3, 7, 10]. Alternativ können die Arterien auch durch permanente Embolisate in Form von Mikrosphären verschlossen werden [3, 7]. Die Embolisation führt zu einem vorübergehenden bzw. permanenten Verschluss der das Gelenk umgebenden Arterien [7]. Dadurch werden die lokale Hypervaskularisation und inflammatorische Hyperperfusion reduziert – ein Mechanismus, der insbesondere bei aktivierter Arthrose [7], aber auch bei lokalisierter Gelenkentzündung therapeutisch wirksam sein kann.

**Abb. 1:**
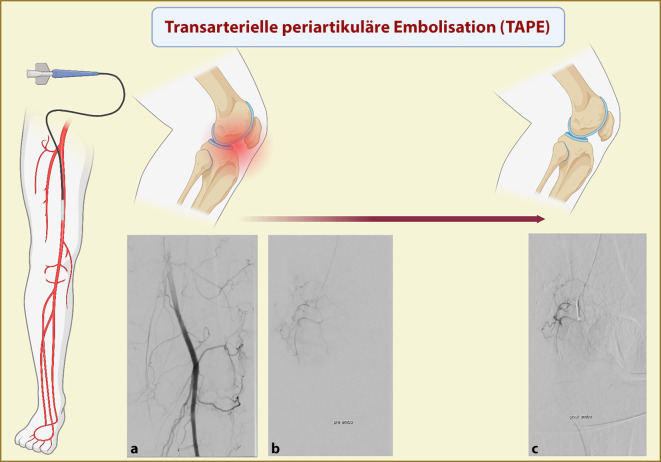
Darstellung der transarteriellen periartikulären Embolisation (TAPE) des Kniegelenks bei Gonarthrose. **a** Angiogramm der Kniegelenkarterien, **b** Angiogramm prä TAPE und **c** Angiogramm post TAPE mit Darstellung der Reduktion der Perfusion der kleinen Kniegelenkarterien. (Agreement number: HY28FQDA7C, created in BioRender. Pfeil, A [2025] https://BioRender.com/htd02gx)

Nach Abschluss der Prozedur wird der Katheter entfernt und die Punktionsstelle entsprechend behandelt. Die Gesamtdauer der Prozedur beläuft sich in der Regel auf 30 Minuten. Neben dem klassischen Zugang über die Leistenschlagader kann auch alternativ über die A. radialis behandelt werden, was lange Liegezeiten und Immobilisation vermeidet (Details siehe Abb. 1).

## Technische und organisatorische Voraussetzungen zur Durchführung der TAPE

Im Folgenden werden die technischen und organisatorischen Voraussetzungen erörtert, die für die Durchführung der TAPE erforderlich sind. Die Durchführung der TAPE erfordert eine interdisziplinäre Zusammenarbeit zwischen Rheumatologie und interventioneller Radiologie. Die Zuweisung zu den Fachbereichen erfolgt dabei entweder über Hausärzt:innen, Orthopäd:innen oder direkt durch die Patient:innen selbst. Die rheumatologische Erstabklärung umfasst eine Anamnese, eine klinische Untersuchung, eine Sichtung der Vorbefunde sowie ggf. eine ergänzende Bildgebung (Röntgen, Arthrosonographie, MRT) und Labordiagnostik.

Aus technischer Perspektive ist eine angiographiefähige Anlage zur Darstellung peripherer Gefäße erforderlich. Die Intervention wird durch Fachärzt:innen der interventionellen Radiologie durchgeführt. Nach einer interdisziplinären Indikationsstellung erfolgt die stationäre Aufnahme, Aufklärung und Durchführung der TAPE.

## TAPE zur Behandlung der Gonarthrose

Ursprünglich wurde die genannte Therapieform zur Behandlung der Gonarthrose entwickelt [14], wobei das Verfahren eine signifikante Schmerzreduktion durch gezielte temporäre Modulation der Durchblutung periartikulärer Gefäßstrukturen zeigt [14].

Derzeit liegt der primäre Anwendungsbereich in der Behandlung der Gonarthrose. Okuno et al. publizierten erste Daten zur TAPE bei einer Gonarthrose. Die Auswertung der erhobenen Daten mittels des „Western Ontario and McMaster Universities Osteoarthritis Index pain scores“ (WOMAC-Score) ergab eine signifikante Reduktion des Schmerzes. Konkret wurde von einem Wert von 12,1 (Baseline) auf 2,6 (Monat 24 post TAPE) ein Fortschritt verzeichnet. Die Erfolgsrate der Embolisation betrug 86,3 % nach einer Nachbeobachtungszeit von 36 Monaten. In der vorliegenden Studie wurde keine Osteonekrose oder Progression der Gonarthrose festgestellt [14]. Ein gleichsinniges Resultat konnte in 4 weiteren Studien für die milde bis moderate Gonarthrose beschrieben werden [2, 8, 12, 17], wenngleich bei einer schweren Gonarthrose nach initialer Schmerzreduktion es nach Monat 3 nach einer TAPE zu einem Anstieg des Schmerzes auf das Ausgangsniveau zu verzeichnen war [12]. Hinsichtlich der Anwendung temporärer vs. permanenter Embolisate zur Reduktion von Schmerzen konnten in einer kleinen Fallserie keine signifikanten Unterschiede beobachtet werden [4].

Die Reduktion des Schmerzes korrelierte mit einer geringeren Einnahme von Analgetika, insbesondere nichtsteroidalen Antirheumatika [12]. Die Wirksamkeit der Behandlung wurde vonseiten der Patienten mit exzellent (12 %) bzw. gut (41 %) eingeschätzt [8]. Zusätzlich zeigt die Knochenmineraldichte im Bereich der proximalen Tibia nach einer TAPE keine signifikante Veränderung über einen Zeitraum von 6 Monaten [8].

In einer kleinen Studie zur TAPE konnte nach Implantation einer Knieendoprothese eine signifikante Reduktion der Kniegelenkschmerzintensität (Baseline: 73 ± 16 mm vs. Monat 6: 38 ± 35 mm) sowie eine Verbesserung des Knee Injury and Osteoarthritis Outcome Score (KOOS, Baseline: 43,6 ± 15,5 Punkte vs. Monat 6: 64,6 ± 27,1 Punkte) nachgewiesen werden [6]. Der KOOS ist ein strukturierter Fragebogen, der dazu dient, die Schmerzintensität, die Symptome, die Aktivitäten des täglichen Lebens, die Funktion bei sportlichen Aktivitäten und die kniespezifische Lebensqualität nach Knieverletzungen und bei einer Gonarthrose zu erfassen [16].

## Weitere Zielregionen zum Einsatz der TAPE

Darüber hinaus wurden vielversprechende Einzelfallberichte und kleinere Studien zur erfolgreichen Embolisation bei Fingerarthrosen und Schulterschmerzen publiziert, was die Übertragbarkeit des Verfahrens auf weitere Gelenkregionen nahelegt. In einer kleinen Studie konnte die Anwendung von TAPE zur Behandlung einer Rhizarthrose über einen Zeitraum von 24 Monaten hinweg eine signifikante Reduktion des Schmerzes bei einer hundertprozentigen Erfolgsrate ohne Nachweis von Nebenwirkungen nachweisen [9]. In der wissenschaftlichen Literatur finden sich Fallberichte zu TAPE des Schultergelenks, des Ellenbogengelenks und einzelner kleiner Fingergelenke [10, 18].

## Potenzielle Einsatzgebiete in der Rheumatologie

Neben der Anwendung bei degenerativen Gelenkveränderungen bietet die TAPE ein potenzielles neues Verfahren als rheumatologisch interventioneller Therapieansatz.

Insbesondere bei persistierender Monarthritis, die trotz systemischer Basistherapie nicht vollständig regressiert, kann die TAPE eine lokal begrenzte, komplementäre Maßnahme darstellen. Zudem weisen erste Erfahrungen auf eine potenzielle Wirksamkeit bei therapieresistenter Arthritis im Kontext immunonkologischer Therapien hin, insbesondere bei der Checkpointinhibitor-assoziierten Arthritis [15]. In diesen Fällen stellt die TAPE eine vielversprechende Option dar, um lokale Inflammation gezielt zu modulieren, ohne die systemische Immunkontrolle zu kompromittieren.

Das Potenzial des Verfahrens liegt in der Evaluierung der Möglichkeit der lokalen, hochkonzentrierten Applikation von Medikamenten mittels TAPE im Bereich einzelner entzündlich veränderter Gelenke.

## Sicherheit und Komplikationen

Bei Anwendung temporärer Embolisate, wie z. B. Cilastatin/Imipenem, wurde in 2,5 % ein transientes kutanes Hautödem beschrieben [7]. Die Verwendung permanenter Embolisate (z. B. Mikrosphären) ist hingegen mit einem erhöhten Risiko für temporäre Hautirritationen (12 %) assoziiert [7]. Die Wahl des Embolisats ist somit entscheidend für die Sicherheit und Verträglichkeit des Verfahrens (Details s. Tab. 1). Zu beachten ist jedoch, dass die Verwendung von Cilastatin/Imipenem im Rahmen der TAPE einen Off-label-Use darstellt und diesbezüglich ein gesondertes Einverständnis im Rahmen der Aufklärung einzuholen ist.

**Tab. 1:** Mögliche Komplikation der transarteriellen periartikulären Embolisation, differenziert nach temporären und permanenten Embolisaten. (Adaptiert nach Heller et al. [7])

	Temporäre Embolisate (z. B. Cisplatin/Imipenem)	Permanente Embolisate (z. B. Mikrosphären)
Transientes kutanes Hautödem	2,5 % (Woche 1–3)	12 % (zwischen Monat 1 und 3: 63 %)
Hämatome im Zugangsbereich (%)	10	
Plantare sensorische Parästhesien (%)	1,1	
Fieber (%)	0,55	

## Ausblick und potenzielle Limitationen

Um die Effektivität des Verfahrens zu untersuchen, sind randomisierte klinische Studien durchzuführen. Es wird empfohlen, diese auch unter dem Gesichtspunkt der Reduktion des Schmerzes, der Einnahme von Schmerztherapeutika (z. B. nichtsteroidale Antirheumatika) bzw. der Evaluation der Verzögerung des Zeitpunkts der Implantation eines endoprothetischen Gelenkersatzes, beispielsweise bei einer Gonarthrose, als unter auch Gesichtspunkten der Lebensqualität durchzuführen.

Die Evaluation der Langzeitwirkung einer TAPE ist ein weiterer wesentlicher Aspekt, da sie von entscheidender Bedeutung für die Implikationen des Verfahrens in die klinische Routine ist. Es ist zu untersuchen, ob im Vergleich zur Radiosynoviorthese als mögliches konkurrierendes und lokales Therapieverfahren eine bessere Behandlung des einzelnen Gelenks möglich ist, da für die Radiosynoviorthese nur eine temporäre Wirkung für maximal 6 Monate in der Literatur beschrieben wird [1, 5]. An dieser Stelle sei angemerkt, dass ggf. auch weitere vergleichende Studien zur Radiosynoviorthese durchgeführt werden sollten, da ein behandeltes arthritisches Gelenk bei einer rheumatoiden Arthritis mittels Radiosynoviorthese nur eine Ansprechrate im Mittel von 50 % aufweist [13]. Gemäß einer aktuellen Studie, die den Einsatz der Radiosynoviorthese bei einer Monarthritis im Rahmen einer rheumatoiden Arthritis untersucht, treten bei 54,8 % der Fälle Schwellungen und bei 51,6 % der Fälle Schmerzen postinterventionell im Bereich des behandelten Knies auf [19]. Im Rahmen einer Radiosynoviorthese wird zudem eine Hautnekrose bzw. Knochennekrose beschrieben [1].

Weiterführend sollte in klinischen Studien Zur TAPE geprüft werden, welches Embolisat die beste Wirksamkeit aufweist und ob es bei der Wahl der Embolisate Unterschiede zwischen einzelnen Gelenkgruppen gibt. Im vorliegenden Kontext gilt es ebenfalls, Langzeitsicherheitsdaten für die TAPE zu erheben.

## Zusammenfassung

Die transarterielle periartikuläre Embolisation (TAPE) stellt ein innovatives, interventionelles Verfahren mit hohem Potenzial für die Schmerztherapie bei Arthrose dar – und eröffnet zugleich neue Perspektiven für den gezielten Einsatz bei entzündlich rheumatischen Gelenkerkrankungen. Angesichts vielversprechender erster Erfahrungen bietet die TAPE eine spannende Erweiterung des therapeutischen Arsenals im Bereich der Rheumatologie.

## Fazit für die Praxis

- Die transarterielle periartikuläre Embolisation (TAPE) stellt ein vielversprechendes minimal-invasives Verfahren zur Behandlung chronischer Gelenkschmerzen dar, das insbesondere bei milder bis moderater Gonarthrose Anwendung finden kann.
- Die vorliegenden ersten klinischen Daten legen eine signifikante und anhaltende Reduktion von Schmerzen, eine geringere Einnahme von Analgetika sowie eine hohe Patientenzufriedenheit nahe.
- Auch bei persistierenden Monarthritiden, etwa im Rahmen entzündlich rheumatischer Gelenkerkrankungen oder immunvermittelter Nebenwirkungen (z. B. Checkpointinhibitor-assoziierte Arthritis), eröffnet die TAPE eine neue, lokal wirksame Therapieoption.
- Für eine weitere Etablierung sind randomisierte, kontrollierte Studien erforderlich, um den Stellenwert von TAPE im Vergleich zu konservativen und operativen Verfahren sowie ihren möglichen Einsatz bei entzündlich rheumatischen Gelenkerkrankungen systematisch zu evaluieren.

## Abkürzungen

KOOS: Knee Injury and Osteoarthritis Outcome Score
TAPE: Transarterielle periartikuläre Embolisation
VAS: Visuelle Analogskala
